# Combination Effect of Antituberculosis Drugs and Ethanolic Extract of Selected Medicinal Plants against Multi-Drug Resistant *Mycobacterium tuberculosis* Isolates

**DOI:** 10.3390/scipharm85010014

**Published:** 2017-03-20

**Authors:** Prabasiwi Nur Fauziyah, Elin Yulinah Sukandar, Dhyan Kusuma Ayuningtyas

**Affiliations:** School of Pharmacy, Bandung Institute of Technology, Bandung, West Java 40132, Indonesia; elin@fa.itb.ac.id (E.Y.S.); dhyankusumaayuningtyas@gmail.com (D.K.A.)

**Keywords:** combination, *Hibiscus sabdariffa*, *Kaempferia galanga*, *Piper crocatum*, antituberculosis drug

## Abstract

Adverse drug reaction and resistance to antituberculosis drugs remain the causes of tuberculosis therapeutic failure. This research aimed to find the combination effect of standard antituberculosis drugs with *Hibiscus sabdariffa* L., *Kaempferia galanga* L., and *Piper crocatum* N.E. Br against multi-drug resistant (MDR) *Mycobacterium tuberculosis* isolates. Two MDR strains (i.e., isoniazid/ethambutol resistant and rifampicin/streptomycin resistant) of *M. tuberculosis* were inoculated in Löwenstein–Jensen medium containing a combination of standard antituberculosis drugs and ethanolic extracts of *H. sabdariffa* calyces, *K. galanga* rhizomes, and *P. crocatum* leaves using various concentration combinations of drug and extract. The colony numbers were observed for 8 weeks. The effect of the combination was analyzed using the proportion method which was calculated by the mean percentage of inhibition reduction in a number of colonies on drug–extract containing medium compared to extract-free control medium. The results showed that all three plant extracts achieved good combination effects with rifampicin against the rifampicin/streptomycin resistant strain. Antagonistic effects were, however, observed with streptomycin, ethambutol and isoniazid, therefore calling for caution when using these plants in combination with antituberculosis treatment.

## 1. Introduction

Tuberculosis (TB) remains one of the deadliest communicable diseases globally, including in Indonesia. The World Health Organization (WHO) estimated that globally in 2013, there were approximately 9 million people suffering from tuberculosis and 1.5 million died from tuberculosis, 360,000 of whom were TB-human immunodeficiency virus (HIV) positive. About 56% of TB patients in the world were in the South East Asia region and West Pacific region.

Although strategies to eradicate tuberculosis have been applied worldwide, tuberculosis eradication is still facing many problems and more development in strategic therapy is required. Increasing cases of multi-drug resistant tuberculosis (MDR-TB) and poor achievement in therapeutic goals are the major concerns. The proportion of new MDR-TB cases was 3.5% of all new tuberculosis cases globally and the number of MDR-TB patients increased three-fold in 2013. It was estimated that 6800 new cases of MDR-TB were developed in Indonesia and more than 55% of MDR-TB patients were not correctly diagnosed or treated [1]. The length of therapy also remains a burden. Tuberculosis patients need two months of intensive phase therapy followed by four months of maintenance phase therapy. Many patients did not achieve therapeutic the therapeutic goals due to side effects during therapy and non-compliance with the regimen of therapy [2].

The need to accelerate the eradication of tuberculosis globally requires more inventions and developments. Standard antituberculosis drugs used today were introduced 40 or more years ago [3]. The world urgently needs new regimens that can shorten and simplify the treatment, that are active against multi-drug strains and can be administrated with antiretroviral drugs to improve therapeutic outcomes.

Medicinal plants offer a big chance to meet the needs of new antituberculosis regimens. A large number of plant species have been investigated for their activity against *Mycobacterium tuberculosis*. More recently, several studies have reported that native Indonesian plants that are traditionally used for respiratory diseases have specific antimycobacterial activity [4,5,6]. Nevertheless, using medicinal plants or natural products as a single agent to treat tuberculosis is not recommended by health professionals because of the rising threat of resistance to inadequate treatment. Besides that, there is no study reporting that a combination of antituberculosis drugs and medicinal plant extracts improve outcome. Therefore, this study aimed to demonstrate the activity of standard antituberculosis drugs combined with *Hibiscus sabdariffa* L., *Kaempferia galanga* L., and *Piper crocatum* N.E. Br against multi-drug resistant (MDR) *M. tuberculosis* isolates.

## 2. Materials and Methods

### 2.1. Plant Collection

*H. sabdariffa* L., *K. galanga* L., and *P. crocatum* N.E. Br used for this study were obtained from Manoko Botanical Garden, Lembang, Bandung, Indonesia and identified at School of Life Sciences and Technology, Bandung Institute of Technology. The dried calyces of *H. sabdariffa* L., dried rhizomes of *K. galanga* L., and dried leaves of *P. crocatum* N.E. Br were cut into small pieces and powdered mechanically and stored in a sealed container.

### 2.2. Extraction

Each dried powdered plant was extracted using the reflux method and ethanol as solvent. The solvent was replaced twice, every 3 hours from the solvent boiling time. The liquid ethanolic extracts obtained were filtered and concentrated using a rotary evaporator (Buchi Labortechnik AG, Postfach, Flawil, Switzerland) for approximately 3 weeks until no ethanol dropped into the solvent flask on the rotary evaporator. The extracts were further concentrated using a waterbath set at 80 °C. These two steps of concentration will eliminate the ethanol residue in the extracts. The concentrated extracts obtained were weighted and stored in an airtight container.

### 2.3. *Mycobacterium* Strain

This study used two isolated strains of multi-drug resistant *M. tuberculosis,* i.e., rifampicin/streptomycin resistant and isoniazid/ethambutol resistant. *Mycobacterium* isolates used for this study must be 2–4 weeks old. The bacteria strains were provided by Department of Microbiology, Health Laboratory of West Java Province, Bandung, Indonesia. The drug-resistant strains were isolated and cultured from a patient with MDR TB, but the TB treatment history of the patient was unknown because the patient treatment history was at a different health facility.

### 2.4. Preliminary Study

The extracts were diluted using sterile water and incorporated to medium to attain concentrations of 50, 100, 250, 500, 750, and 1000 µg/mL. The results of this step were used to determine the extract concentration to be combined with antituberculosis drugs. Extract-free medium with bacteria was used as a positive control and extract-free medium without bacteria was used as a negative control. These extracts and controls were duplicated to increase data reliability. The previously prepared bacteria were inoculated to each medium. All samples were incubated at 37 °C for 8 weeks. The number of colonies was counted every week.

### 2.5. Drug–Extract Combination Assay

The test was performed as an antimycobacterial activity assay using the Löwenstein–Jensen (LJ) proportion method with slight modification [7]. The standard extract concentration used in this method was 1000 µg/mL for *H. sabdariffa* and *P. crocatum*, and 500 µg/mL for *K. galanga*. The standard antituberculosis drug concentrations used in this method were 40 µg/mL, 4 µg/mL, 2 µg/mL, and 0.2 µg/mL for rifampicin, streptomycin, ethambutol, and isoniazid, respectively. Two series of stock solution were made by diluting each extract and each antituberculosis standard drug with sterile distilled water. The concentrations of stock solution used were 30 mg/mL and 15 mg/mL for extract stock solutions and 120 µg/mL and 60 µg/mL for standard drug stock solutions. To make the 40:1000 combination of rifampicin and *H. sabdariffa* extract, 2 mL of 30 mg/mL extract stock solution and 20 mL of 120 µg/mL rifampicin stock solution were added to 38 mL of sterile LJ medium solution and shaken gently until homogenous. To make the 4:1000 combination of streptomycin and *H. sabdariffa* extract, 2 mL of 30 mg/mL extract stock solution and 2 mL of 120 µg/mL streptomycin stock solution were added to 56 mL of sterile LJ medium solution and shaken gently until homogenous. To make the 2:1000 combination of ethambutol and *H. sabdariffa* extract, 2 mL of 30 mg/mL extract stock solution and 1 mL of 120 µg/mL ethambutol stock solution were added to 57 mL of sterile LJ medium solution and shaken gently until homogenous. To make the 0.2:1000 combination of isoniazid and *H. sabdariffa* extract, 2 mL of 30 mg/mL extract stock solution and 0.10 mL of 120 µg/mL ethambutol stock solution were added to 57.90 mL of sterile LJ medium solution and shaken gently until homogenous. The same procedure was repeated to make the combination of *P. crocatum* extract–standard drugs. To make the combination of *K. galanga* extract and standard drugs, the procedure was similar to the previous procedure but the extract volume stock added was 1 mL and the media volume was adjusted to 60 mL total volume. The procedure to make the combination with a lower concentration of extracts and drugs was similar to the previous combination procedure, but used the 15 mg/mL extract stock solutions and 60 µg/mL standard drug stock solutions. Table 1 and Table 2 summarize this preparation step. Drug-containing medium solution and drug–extract-free medium solution, used as controls, were also prepared at the same time.

Every 7 mL of each of extract–drug-medium solution was pipetted and dispensed into a sterile McCartney bottle. These bottles were arranged on a particular rack at 30° lean-position and then incubated at 85 °C for 45 min to coagulate. After incubation time, the LJ-medium bottles were cooled down to room temperature for 24 h before being inoculated with *M. tuberculosis* isolates.

Two strains of MDR *M. tuberculosis* sub-culture isolates were suspended and diluted into 10^−3^ and 10^−5^ McFarland using 5-fold dilution step methods from a 1.0 McFarland standard dilution. An amount of 100 µL of rifampicin/streptomycin resistant strain mycobacterial suspension of 10^−3^ McFarland dilution was inoculated to each drug–extract containing medium bottle and drug-containing medium bottle, except the negative control medium bottle. The same procedure was repeated to the isoniazid/ethambutol resistant strain and 10^−5^ McFarland concentration. After inoculation, all the medium bottles were incubated at 37 °C. The number of colonies of MDR *M. tuberculosis* was observed and counted every week for 8 weeks.

## 3. Results

As the study was performed as an antimycobacterial assay, the data collected were *Mycobacterium* colony counts on LJ medium for 8 weeks. The collected data were then processed using the proportion method which resulted in a percentage of inhibition as shown in Table 3 for the preliminary study and Table 4 for the drug–extract combination study.

From the preliminary study, the inhibition of *M. tuberculosis* growth for all the medicinal plants used was more than 70% ± 0.0%, even at the lowest concentration. A concentration of 500 µg/mL was used as the standard concentration of *K. galanga* extract for the combination study because it was the lowest concentration of *K. galanga* giving 100% inhibition. A concentration of 1000 µg/mL was used as the standard concentration of *H. sabdariffa* and *P. crocatum* extract for the combination study because it was the highest concentration giving *M. tuberculosis* growth inhibition; the total inhibition (100%) could not be met by any of the extract concentrations used. Although the results did not follow a dose–response relationship, choosing 1000 µg/mL as the standard concentration for *H. sabdariffa* and *P. crocatum* was thought to be the safest way to avoid inadequate inhibition activity in the combination study.

The results of this study showed that, for the rifampicin/streptomycin resistant strain, an additive effect was obtained when combining rifampicin with all three extracts. In particular, *P. crocatum* showed the highest increase in the percentage of inhibition, rising from 44% to 100% within the combination, even at the lower dose of 500 µg/mL. Regarding the other antibiotics however, a decrease in the potency was observed, particularly for the combination with *K. galanga*. Indeed, the extract alone displayed 100% growth inhibition at 500 µg/mL, while its combination with streptomycin, ethambutol and isoniazid yielded only 55%, 76% and 50% inhibition, respectively.

## 4. Discussion

Current tuberculosis treatment is facing major problems such as complexity and lengthy treatment duration. Adverse reactions to antituberculosis drugs may lead to non-adherence to treatment, causing poor outcomes and also therapeutic failure [8]. Another major concern is the increasing incidence of MDR tuberculosis, not only to the first-line antituberculosis drugs isoniazid and rifampicin, but also to the second-line drugs that are now becoming more common. Strengthening current tuberculosis treatment and discovering new antituberculosis drugs are required to overcome the challenges and to reach the WHO 2035 goal of ending the global tuberculosis epidemic [1,8,9]. The plants selected in this study are all used in Indonesian traditional medicine to treat respiratory disorders and have also been studied for their activity against *M. tuberculosis*.

In the present work, all of the selected medicinal plants used gave good combination effects with rifampicin, as compared to the drug used alone. The presence of the extract of three selected medicinal plants on LJ medium along with the antituberculosis drug could increase antimycobacterial activity against the rifampicin/streptomycin clinical isolates. However, *K. galanga*, *P. crocatum*, and *H. sabdariffa* had antagonistic effects with streptomycin, ethambutol and isoniazid. This was particularly apparent for *K. galanga*, which was able to fully inhibit the growth of both drug-resistant isolates alone, but lost this capacity when combined with antituberculosis drugs.

This study is the first to report on the combination effect of *K. galanga*, *P. crocatum*, and *H. sabdariffa* with standard antituberculosis drugs since previous studies have only investigated their antimycobacterial activity as a single agent. A study in India showed that Ethyl *p*-Methoxycinnamate (EPMC) isolated from *K. galanga* inhibited the MDR strain of *M. tuberculosis*. Ethyl cinnamate and EPMC were the major and vital constituents in the *K. galanga* extract, suspected to have antimycobacterial activity besides its larvacidal, nematicidal, anti-oxidant, antimicrobial, antineoplastic, and anti-inflammatory activity [10,11]. EPMC seems to be leading molecule to develop a new potent antituberculosis drug. In our study, it is still unknown whether the antimycobacterial mechanism of EPMC has the same molecule target or same mechanism of action as the standard antituberculosis drug.

Our results were in agreement with a previous in vitro and in vivo study using ethanolic extract of *P. crocatum* as an antimycobacterial agent, which revealed that exposure of the *P. crocatum* ethanolic extract can decrease the *M. tuberculosis* level both in vitro and in vivo [4]. The phytochemicals contained in *P. crocatum* were alkaloids, flavonoid, glycoside, saponin, tannin, and triterpenoid [12]. There were only limited studies about *P. crocatum*, its chemical constituents and activity as an antibacterial agent. None of those studies isolated the phytochemical compound of *P. crocatum* and investigated its activity against *M. tuberculosis*. Cell wall destruction was predicted as the mechanism of action of *P. crocatum* against *M. tuberculosis*. An observation under transmission electron microscope showed damage on the cell wall of *M. tuberculosis* after exposure to ethanolic extract of *P. crocatum* [4].

Our current finding on the combination effect of *H. sabdariffa* was also consistent with a previous study which showed that extract of *H. sabdariffa* inhibited the MDR strain of *M. tuberculosis* [5]. Constituents such as organic acid (hibiscus acid, hibiscus acid glucoside, hydroxycitric acid), anthocyanins (delphinidin-3-sambubioside, cyanidin-3-sambubioside), and flavonoids and phenolic acid (gallic acid, quercetin, myricetin, kaempferol, protocatechuic acid, etc.) were the main phytochemical compounds of *H. sabadariffa* related to its activity [13,14]. The exact mechanism of action against *M. tuberculosis* is not fully understood. An increase in plasma membrane permeability following inhibition of various cellular processes, leading to ion leakage, was predicted as the antibacterial mechanism of action of *H. sabdariffa* [15].

## 5. Conclusions

The results of our study indicated that *K. galanga*, *P. crocatum*, and *H. sabdariffa* gave good combination effects with rifampicin to combat the rifampicin/streptomycin resistant *M. tuberculosis* strain. However, an antagonistic effect was shown when all of the three extracts were combined with streptomycin, ethambutol, and isoniazid. The main mechanism of action is still unclear and uncertain as further studies needed. Future studies need to address the main pharmacologically active compound as an antimycobacterial agent and the target of the antituberculosis active compound in order to support the in vitro study result. In vivo studies are also needed to observe their effectiveness in whole-organism systems including toxicity studies. Use of these medicinal plants as traditional remedies in combination with antituberculosis drugs should be done with caution as not all plants displayed good synergic combination effects.

## Figures and Tables

**Table 1:** Summary of the preparation to make the *Hibiscus sabdariffa* and *Piper crocatum* combination with standard antituberculosis drugs.

Standard Drug	Standard Drug Stock Concentration (µg/mL)	Drug Stock Volume (mL)	Extract Stock Concentration (µg/mL)	Extract Stock Volume (mL)	Media Volume (mL)	Total Volume (mL)	Final Drug:Extract Ratio Concentration
Rifampicin	120	20.00	30,000	2.000	38.00	60	40:1000
Isoniazid	120	0.10	30,000	2.000	57.90	60	0.2:1000
Ethambutol	120	1.00	30,000	2.000	57.00	60	2:1000
Streptomycin	120	2.00	30,000	2.000	56.00	60	4:1000
Rifampicin	60	20.00	15,000	2.000	38.00	60	20:500
Isoniazid	60	0.10	15,000	2.000	57.90	60	0.1:500
Ethambutol	60	1.00	15,000	2.000	57.00	60	1:500
Streptomycin	60	2.00	15,000	2.000	56.00	60	2:500

**Table 2:** Summary of the preparation to make the *Kaempferia galanga* combination with standard antituberculosis drugs.

Standard Drug	Standard Drug Stock Concentration (µg/mL)	Drug Stock Volume (mL)	Extract Stock Concentration (µg/mL)	Extract Stock Volume (mL)	Media Volume (mL)	Total Volume (mL)	Final Drug:Extract Ratio Concentration
Rifampicin	120	20.00	30,000	1.00	39.00	60	40:500
Isoniazid	120	0.10	30,000	1.00	58.90	60	0.2:500
Ethambutol	120	1.00	30,000	1.00	58.00	60	2:500
Streptomycin	120	2.00	30,000	1.00	57.00	60	4:500
Rifampicin	60	20.00	15,000	1.00	39.00	60	20:250
Isoniazid	60	0.10	15,000	1.00	58.90	60	0.1:250
Ethambutol	60	1.00	15,000	1.00	58.00	60	1:250
Streptomycin	60	2.00	15,000	1.00	57.00	60	2:250

**Table 3:** Percent of inhibition of various extract concentrations against multi-drug resistant (MDR) *Mycobacterium tuberculosis* isolates.

Botanical Name	Concentration (µg/mL)	*Mycobacterium tuberculosis* Strain							
		HE^a^ Resistant				RS^b^ Resistant			
		1	2	Mean CFU^c^	Mean % Inhibition	1	2	Mean CFU^c^	Mean % Inhibition
Control (-)	-	0	0	0 ± 0	-	0	0	0 ± 0	-
Control (+)	-	390	176	283 ± 107	-	216	375	295 ± 80	-
*Hibiscus sabdariffa* L.	50	81	60	71 ± 10	73 ± 7	131	109	120 ± 11	55 ± 16
	100	64	56	60 ± 4	76 ± 8	101	105	103 ± 2	63 ± 9
	250	71	82	76 ± 6	68 ± 14	134	151	143 ± 9	49 ± 11
	500	72	76	74 ± 2	69 ± 12	132	112	122 ± 10	54 ± 16
	750	84	82	83 ± 1	66 ± 12	105	105	105 ± 0	62 ± 10
	1000	11	44	27 ± 17	86 ± 11	134	117	126 ± 9	53 ± 15
*Kaempferia galanga* L.	50	85	43	64 ± 21	77 ± 1	176	63	120 ± 56	51 ± 32
	100	62	33	47 ± 15	83 ± 1	129	115	122 ± 7	55 ± 14
	250	24	9	17 ± 7	94 ± 0	16	15	16 ± 1	94 ± 2
	500	6	0	3 ± 3	99 ± 1	0	0	0 ± 0	100 ± 0
	750	0	0	0 ± 0	100 ± 0	0	0	0 ± 0	100 ± 0
	1000	0	0	0 ± 0	100 ± 0	0	0	0 ± 0	100 ± 0
*Piper crocatum* N.E. Br.	50	41	41	41 ± 0	83 ± 6	144	144	144 ± 0	47 ± 14
	100	53	65	59 ± 6	75 ± 12	125	153	139 ± 14	51 ± 8
	250	54	56	55 ± 1	77 ± 9	116	124	120 ±	57 ± 10
	500	38	51	44 ± 6	81 ± 10	148	159	154 ± 5	44 ± 13
	750	56	46	51 ± 5	80 ± 6	149	165	157 ± 8	43 ± 12
	1000	39	38	38 ± 0	84 ± 6	152	152	152 ± 0	44 ± 15

^a^ Isoniazid-Ethambutol; ^b^ Rifampicin-Streptomycin; ^c^ Colony Forming Unit.

**Table 4:** The percentage of inhibition for various combinations of antituberculosis drug and extract against MDR Mycobacterium tuberculosis isolates.

Standard Drugs	Extract	Concentration in Combination (µg/mL)	*Mycobacterium tuberculosis* Strain							
			HE Resistant				RS Resistant			
			1	2	Mean CFU	Mean % Inhibition	1	2	Mean CFU	Mean % Inhibition
Control (+)	-	-	128	188	158 ± 30	-	173	179	176 ± 3	-
Rifampicin	-	-	0	0	0	100 ± 0	148	118	133 ± 15	24 ± 10
	*H. sabdariffa*	40:1000	0	0	0	100 ± 0	0	0	0	100 ± 0
	*P. crocatum*	40:1000	0	0	0	100 ± 0	0	0	0	100 ± 0
	*K. galanga*	40:500	0	0	0	100 ± 0	0	0	0	100 ± 0
	*H. sabdariffa*	20:500	0	0	0	100 ± 0	0	0	0	100 ± 0
	*P. crocatum*	20:500	0	0	0	100 ± 0	0	0	0	100 ± 0
	*K. galanga*	20:250	0	0	0	100 ± 0	0	0	0	100 ± 0
Streptomycin	-		0	0	0	100 ± 0	128	222	175 ± 47	1 ± 1
	*H. sabdariffa*	4:1000	0	0	0	100 ± 0	141	129	135 ± 6	23 ± 5
	*P. crocatum*	4:1000	0	0	0	100 ± 0	127	117	122 ± 5	31 ± 4
	*K. galanga*	4:500	0	0	0	100 ± 0	98	58	78 ± 20	55 ± 12
	*H. sabdariffa*	2:500	0	0	0	100 ± 0	121	103	112 ± 9	36 ± 6
	*P. crocatum*	2:500	0	0	0	100 ± 0	79	79	79 ± 0	55 ± 1
	*K. galanga*	2:250	0	0	0	100 ± 0	52	124	88 ± 36	50 ± 20
Ethambutol	-	-	181	130	156 ± 26	1 ± 3	0	0	0	100 ± 0
	*H. sabdariffa*	2:1000	123	121	122 ± 1	20 ± 16	0	0	0	100 ± 0
	*P. crocatum*	2:1000	88	88	88 ± 0	42 ± 11	0	0	0	100 ± 0
	*K. galanga*	2:500	48	17	33 ± 15	76 ± 14	0	0	0	100 ± 0
	*H. sabdariffa*	1:500	130	26	78 ± 52	55 ± 24	0	0	0	100 ± 0
	*P. crocatum*	1:500	98	93	96 ± 3	37 ± 14	0	0	0	100 ± 0
	*K. galanga*	1:250	140	86	113 ± 7	29 ± 4	0	0	0	100 ± 0
Isoniazid	-	-	223	181	202 ± 21	0 ± 0	0	0	0	100 ± 0
	*H. sabdariffa*	0.2:1000	133	137	135 ± 2	15 ± 12	0	0	0	100 ± 0
	*P. crocatum*	0.2:1000	109	103	106 ± 3	30 ± 15	0	0	0	100 ± 0
	*K. galanga*	0.2:500	74	84	79 ± 5	50 ± 7	0	0	0	100 ± 0
	*H. sabdariffa*	0.1:500	140	129	135 ± 5	20 ± 11	0	0	0	100 ± 0
	*P. crocatum*	0.1:500	101	109	105 ± 4	32 ± 10	0	0	0	100 ± 0
	*K. galanga*	0.1:250	80	84	82 ± 2	46 ± 9	0	0	0	100 ± 0
